# Early Diagnosis of Iatrogenic Post-Traumatic Trigeminal Neuropathies: From Clinical Gaps to Medico-Legal Implications—A Comprehensive Structured Narrative Review

**DOI:** 10.3390/jcm15186941

**Published:** 2026-09-08

**Authors:** Alfonso Acerra, Francesco Giordano, Massimo Amato, Iman Rizki, Alessandro Santurro

**Affiliations:** Department of Medicine, Surgery and Dentistry, University of Salerno, 84081 Baronissi, SA, Italy; frgiordano@unisa.it (F.G.); mamato@unisa.it (M.A.); i.rizki@studenti.unisa.it (I.R.); asanturro@unisa.it (A.S.)

**Keywords:** nerve injury, post-traumatic trigeminal neuropathy, inferior alveolar nerve, lingual nerve, early diagnosis, narrative review

## Abstract

**Background**: Iatrogenic injuries of the inferior alveolar nerve (IAN) and lingual nerve (LN) are among the most relevant complications in oral surgery. Although most cases show spontaneous recovery, a significant proportion of patients develop persistent post-traumatic trigeminal neuropathies (PTNs). The aim of this narrative review is to critically analyze current diagnostic approaches for IAN and LN injuries, highlighting the limitations of conventional assessment tools and evaluating the potential role of emerging neurophysiological and imaging techniques in improving early diagnosis, prognostic stratification and medico-legal management. **Methods**: A structured narrative review was conducted using PubMed/MEDLINE, Scopus and Web of Science for English-language studies published up to 23 March 2026. Evidence on trigeminal nerve injuries, neurosensory assessment, quantitative sensory testing (QST), advanced imaging techniques and medico-legal implications was qualitatively synthesized. **Results**: The prognosis of iatrogenic PTNs of both IAN and LN is influenced by the severity of nerve injury and the timing of diagnosis and management. Although conventional neurosensory testing (NST) is widely used in the clinical assessment of trigeminal nerve injuries, its ability to objectively characterize the extent and severity of nerve damage is limited. Complementary neurophysiological and quantitative sensory approaches may provide additional functional information, while magnetic resonance neurography (MRN) offers direct structural assessment of the injured nerve. Early objective identification of nerve injury may support prognostic evaluation and timely clinical decision-making. **Conclusions**: An integrated approach combining clinical assessment, neurophysiology and advanced imaging is essential for early characterization of iatrogenic trigeminal nerve injuries. MRN may provide structural information, contributing to early stratification of injury severity. Quantitative sensory and neurophysiological techniques may complement conventional clinical testing. These diagnostic advances may also have important medico-legal implications, as delayed diagnosis may lead to loss of therapeutic opportunity and increased professional liability.

## 1. Introduction

Among the peripheral branches of the trigeminal nerve that may be affected by iatrogenic procedures, injuries involving the inferior alveolar nerve (IAN) and the lingual nerve (LN) remain common complications in oral and maxillofacial surgery [1,2]. Approximately 60% of neurological damage is associated with dental procedures, specifically the surgical extraction of mandibular third molars, dental implant placement, endodontic treatments complicated by extrusion of materials beyond the apex, and, not least, local anesthetic infiltrations, including direct needle trauma [3,4,5]. To reduce postoperative complications following mandibular third molar extraction or dental implant placement, several therapeutic strategies have been proposed, including autologous platelets concentrates, although their clinical efficacy remains a matter of ongoing investigation [6]. Although many iatrogenic nerve injuries recover spontaneously, a minority of patients develop persistent postoperative neurosensory disturbances, which may range from loss of function to abnormal sensory perception and neuropathic pain, negatively affecting patients’ quality of life [7,8,9].

Current diagnostic approaches rely on chairside neurosensory testing (NST), which has important limitations in objectively characterizing the extent and severity of nerve injury [10,11]. Similarly, radiographic examinations routinely used in clinical practice, including conventional two-dimensional (panoramic radiography) and three-dimensional imaging (Cone Beam Computer Tomography), provide valuable information on bony anatomy but do not allow for direct assessment of the structural integrity of the nerve [8,12].

The limitations of these commonly used diagnostic protocols inevitably may create a temporal gap between the injurious event and prognostic assessment, delaying the timely identification of patients at risk of persistent nerve dysfunction.

The early identification of the severity and extent of nerve injury is therefore clinically relevant, as the timing of appropriate intervention may influence the likelihood of functional recovery, particularly in cases associated with an unfavorable prognosis [13]. Furthermore, immediate assessment of damage in the early postoperative period remains pivotal for selecting the most appropriate treatment plan.

Against this background, the present narrative review critically examines the current evidence on iatrogenic PTN involving IAN and LN, with particular emphasis on the shortcomings of neurosensory testing and conventional imaging, the potential role of emerging diagnostic technologies, and the consequences of delayed diagnosis on treatment timing and medico-legal liability. By integrating clinical, diagnostic and medico-legal perspectives, this review aims to provide a comprehensive framework for the early recognition of severe nerve injuries and informed decision-making in contemporary oral surgery.

## 2. Materials and Methods

### 2.1. Literature Search Strategy

A literature search was conducted using PubMed/MEDLINE, Scopus, and Web of Science databases to identify publications addressing iatrogenic injuries of the inferior alveolar nerve and lingual nerve, with particular emphasis on diagnostic assessment, imaging techniques, prognostic evaluation, microsurgical timing, and medico-legal implications.

Articles published in English from database inception to 23 March 2026 were considered. No restriction regarding publication year was applied. The electronic search combined Medical Subject Headings (MeSH) and free-text terms including:

(“inferior alveolar nerve” OR “lingual nerve” OR “trigeminal nerve”) AND (“injury” OR “damage” OR “neuropathy” OR “paresthesia” OR “dysesthesia”) AND (“diagnosis” OR “diagnostic imaging” OR “clinical evaluation” OR “early diagnosis” OR “prognosis” OR “magnetic resonance neurography” OR “quantitative sensory testing” OR “medicolegal”).

Additional relevant publications were identified through manual screening of the reference lists of selected articles and review papers. The retrieved literature was critically analyzed according to its relevance to the objectives of the present narrative review. Preference was given to the most recent, clinically relevant, and methodologically robust publications, while older landmark studies were included when they provided fundamental concepts regarding nerve injury classification, diagnosis, and treatment. Given the narrative nature of this manuscript and the heterogeneity of the available evidence, no formal systematic review process, PRISMA flow diagram, risk-of-bias assessment, or meta-analysis was performed.

### 2.2. Narrative Synthesis

The narrative synthesis focused on publications addressing one or more of the following topics: etiopathogenesis of inferior alveolar and lingual nerve injuries; classification according to Seddon and Sunderland; clinical manifestations and prognosis; neurosensory assessment; quantitative sensory testing; imaging techniques, including cone beam computed tomography and magnetic resonance neurography; timing of microsurgical intervention; biological mechanisms of nerve degeneration and regeneration; and the medico-legal implications associated with delayed diagnosis and treatment.

Attention was given to studies discussing the diagnostic limitations of currently adopted clinical protocols, the evolution of advanced imaging techniques capable of directly evaluating neural structures, and the relationships between diagnostic delay, therapeutic timing, and functional recovery.

### 2.3. Data Synthesis

The retrieved literature was critically analyzed and organized according to the principal topics addressed in this review. The evidence was synthesized through a thematic approach focusing on the etiopathogenesis of inferior alveolar and lingual nerve injuries, clinical manifestations, neurosensory assessment, conventional and advanced imaging techniques, prognostic evaluation, timing of microsurgical intervention and the medico-legal implications associated with delayed diagnosis and treatment. Attention was given to identifying the limitations of currently adopted diagnostic protocols and to discussing emerging diagnostic strategies that may facilitate earlier recognition of severe nerve injuries and improve clinical decision-making. Owing to the narrative design of this review and the heterogeneity of the available literature, no formal quality assessment, risk-of-bias evaluation, or quantitative meta-analysis was performed.

## 3. Results

The classification of iatrogenic PTNs is based on the well-established histopathological models of Seddon and Sunderland, which provide the framework for correlating the severity of injury with the prognosis for functional recovery [14,15]. Both IAN and LN may sustain different degrees of nerve injury, ranging from transient conduction block to complete axonal disruption [16,17]. The main differences in anatomy, biology, and clinical prognosis between IAN and LN are summarized in Table 1.

### 3.1. Injury Patterns and Mechanisms

With regard to IAN, the literature identifies etiopathogenetic mechanisms closely related to its protected position within the mandibular canal [18]. Libersa et al. and Weckx et al. reported third molar extraction as the most prevalent cause of injury (40.8%), followed by endodontic procedures (35.3%) and implant-related interventions (3.2%) [19,20]. Sarikov and Juodzbalys emphasize that the injury is frequently compressive in nature, caused by displacement of dental roots or the use of surgical elevators, whereas Tay et al. observe that even when the neurovascular bundle appears macroscopically intact during exodontia, a significant risk of paresthesia persists, suggesting the presence of intraneural damage not detectable on gross inspection [1,21]. From an oral surgical perspective, nerve injuries may be clinically apparent intraoperatively, such as in cases of direct transection or severe mechanical trauma, or remain unrecognized during surgery despite macroscopic preservation of nerve continuity, becoming evident only through postoperative neurosensory deficits.

In implantology, injury may manifest as chronic compression due to encroachment of the canal lumen by the fixture or as multiple neurotmetic lesions caused by the drill trajectory during osteotomy preparation [22,23]. Radiographic and clinical evidence has documented cases in which the osteotomy outline overlapped the mandibular canal despite the apparently correct final positioning of the implant [24]. Another type of IAN injury, described by Pogrel et al., concerns chemical toxic lesions from endodontic materials [25]. In particular, extrusion of sodium hypochlorite or root canal sealers beyond the apex may trigger chemical neuritis, described as “axonostenosis” or “axonocachexia,” in which intraneural fibrosis induces secondary ischemia through compression of the vasa nervorum [26].

LN is characterized by markedly greater biological vulnerability due to its oligofascicular configuration, consisting of a few large fascicles surrounded by a thin perineurium and its submucosal course, which lacks osseous protection [27]. Romsa and Ruggiero, together with Lee et al., identify stretch injury as one of the principal mechanisms [28,29]. Iatrogenic retraction of the lingual flap using retractors such as the Howarth may cause internal disruption of the endoneurium or perineurium (Sunderland III–IV) while preserving the macroscopic continuity of the nerve trunk.

A critical morphological difference compared with the IAN is the high incidence of neuroma formation. Zuniga et al. report that neuromas are found in 86% of surgical specimens involving the LN, compared with 43% of those involving the IAN [11]. This prevalence is attributable to the tendency of the severed ends of the LN to retract and become misaligned within soft tissues, triggering disorganized axonal proliferation. Direct needle trauma during truncal anesthesia also plays a relevant etiological role in LN injury. Pogrel and Thamby report that anesthetic block injections account for 20.9% of lingual PTNs, with intraneural needle penetration occurring at approximately twice the rate that observed for the IAN [7]. Patients often describe an “electric shock” sensation during infiltration, a symptom associated with direct needle trauma.

### 3.2. Clinical Symptoms and Outcomes

Post-traumatic trigeminal neuropathy manifests through a complex mosaic of symptoms classified by the International Association for the Study of Pain (IASP) as “negative” and “positive”, ranging from hypoesthesia to paresthesia, dysesthesia, or allodynia [30]. Jääskeläinen et al. report that approximately 45% of patients with stabilized lesions develop chronic neuropathic pain, often described as burning, persistent, or characterized by paroxysmal electric-shock-like sensations [10].

From an anatomo-functional perspective, the sequelae differ depending on the nerve involved. Following IAN injury, the primary consequence is loss of sensitivity of the lower lip, chin, and ipsilateral buccal gingiva.

Lingual nerve injury may result in additional functional impairment because of the involvement of chorda tympani fibers responsible for taste sensation. Cases of ageusia or dysgeusia affecting the anterior two-thirds of the hemilingual surface have been described [2]. Clinical signs associated with severe LN injury (Sunderland Grades IV–V) include mucosal pallor and progressive loss of fungiform papillae, which can be assessed by biomicroscopy [31]. In addition, accidental tongue biting during mastication can occur as a result of sensory impairment with significant repercussions on speech clarity.

In a long-term study of patients monitored between 3 and 9 years after injury, Pogrel et al. documented an incidence of depressive syndrome in 37% of cases, accompanied by difficulties in social and occupational functioning in 20% of subjects [5].

### 3.3. Diagnostic Assessment Approach

The diagnostic workup of iatrogenic trigeminal neuropathies is summarized in Table 2.

The cornerstone of clinical assessment is represented by NST, structured according to the three-level algorithm proposed by Zuniga and Essick [32]. This protocol follows a methodological progression: Level A investigates spatiotemporal perception through two-point discrimination (2PD), directional brush-stroke sensitivity and stimulus localization; Level B evaluates contact threshold using Semmes–Weinstein monofilaments (or von Frey fibers); finally, Level C assesses mechanical nociception (pinprick) and thermal discrimination.

However, the effectiveness of NST is widely debated in the literature. Although Zuniga et al. document high accuracy for the lingual nerve (LN), with a positive predictive value of 95% and a negative predictive value of 100% when the test is normal, the results for the inferior alveolar nerve (IAN) are significantly less efficient [11,33]. For the IAN, the false-negative rate reaches 40%, with a positive predictive value (PPV) of 77% and a negative predictive value (NPV) of 60%, highlighting the limited ability of NST to reliably exclude nerve injury when clinical findings are normal. This limitation is attributable to the intrinsic subjectivity of the method and poor inter-examiner reproducibility.

Quantitative Sensory Testing (QST) provides a non-invasive and standardized psychophysical method, according to the protocols of the German Research Network on Neuropathic Pain (DFNS), capable of quantifying damage to thinly myelinated and unmyelinated fibers (A-δ and C) [34]. Thermal QST, performed with computerized devices such as Peltier thermodes, allows for precise measurement of the cold detection threshold (CDT), warm detection threshold (WDT), and thermal pain thresholds (CPT, HPT). Jääskeläinen et al. report that thermal QST confirms subjective sensory alteration in 91% of patients with IAN-related PTN and in 100% of those with LN involvement, so it may detect persistent deficits even years after trauma, when conventional clinical tests have normalized [10]. In this context, studies by Kim et al. identify loss of warm detection as the most sensitive biomarker, directly correlating with the severity of paresthesia and the onset of neuropathic pain [9,35].

Parallel to this, objectification of the injury can be supported by the measurement of the Current Perception Threshold (CPT) using the Neurometer [36]. This neuroselective electrodiagnostic method directly stimulates axons.

From an imaging perspective, conventional techniques (PAN and CBCT) show a structural information gap in the post-injury phase. In fact, they visualize only the bony “container” (the mandibular canal), identifying cortical interruptions, invading implant fixtures, or foreign materials, but remain “blind” to the neural “content.” Moreover, several authors confirm that CBCT does not statistically reduce the incidence of nerve injuries compared with panoramic imaging [37].

This limitation is overcome by Magnetic Resonance Neurography (MRN) performed with 3.0-Tesla scanners. Dedicated sequences allow for direct visualization of neural tissue, enabling mapping of intraneural architecture and identification of discontinuities, caliber variations and neuromatous formations (terminal or in-continuity neuromas), with a documented specificity of 93.5%. Cassetta et al. reported that measurement of the Relative Signal Intensity (RSI) of the nerve on 3.0-T MRI as early as 3 days after injury may provide an early prognostic indicator [13]. In this prospective study, elevated early RSI values were associated with more severe axonal damage and less favorable recovery.

With regard specifically to the lingual nerve, assessment must include evaluation of chorda tympani function to investigate gustatory deficits. In addition to electrogustometry, the literature proposes chemical taste-stimulation tests which, although simple, are essential for confirming ageusia or dysgeusia associated with high-grade injuries (Sunderland IV–V). Finally, the diagnostic framework may be completed with advanced neurophysiological investigations, such as the blink reflex and somatosensory evoked potentials (SSEP), as adjunctive techniques.

### 3.4. Diagnostic Timing and Intervention

Clinical stabilization of PTNs is generally recommended in the literature to be carried out between 6 and 12 months after the injurious event [38,39]. Beyond this temporal window, in the absence of signs of spontaneous recovery, the injury is considered permanent [40].

An early prognostic assessment can be performed as early as the third postoperative day. Cassetta et al. highlighted that RSI on 3.0-Tesla MRI at this stage could discriminate between lesions associated with spontaneous recovery and those showing delayed or limited recovery [13].

With regard to the IAN, timeliness is particularly critical in implantology. When direct contact or compression of the IAN is suspected, early implant removal or decompression has been recommended within hours or days to prevent the onset of irreversible degenerative processes [39]. For IAN injuries, lack of sensory improvement during the first weeks indicates a less favorable prognosis [7,41].

For LN injuries, the timing of follow-up must be even more stringent, as its spontaneous healing is less probable than that of IAN [42]. Tay and Zuniga suggested that clinical evaluation through NST may provide more reliable prognostic information only after 3 months from the injurious event, a period in which neuropraxic injuries of Grades I and II can be excluded [3]. For high-grade lesions (Sunderland IV–V) microsurgical intervention should be performed between 3 and 6 months after injury. In this context, the meta-analysis by Suhaym and Miloro reported Functional Sensory Recovery (FSR) rates of 93% for repairs performed within 3 months and 78.5% for delayed interventions; although this difference was not statistically significant (*p* = 0.59), the odds of functional improvement significantly favored earlier intervention, with an odds ratio (OR) of 5.49 at the 3-month breakpoint and 2.28 at the 6-month breakpoint [43]. Similarly, other studies have reported a progressive reduction in functional recovery with increasing treatment delay, including a 5.8% reduction in the probability of improvement for each month of delay [44,45].

Beyond 6–12 months, spontaneous recovery becomes less likely and the prognosis of delayed microsurgical repair is generally less favorable [46].

## 4. Discussion

The clinical relevance of iatrogenic PTN derives not only from its frequency but also from the potential persistence and functional burden of sensory disturbances.

The different clinical manifestations and prognostic profiles following IAN and LN injuries can be partly explained by their distinct anatomical and structural characteristics.

The presence of negative and positive sensory symptoms highlights the complexity of PTNs and explains why clinical assessment cannot be reduced to a simple measurement of sensory loss.

Chronic neuropathic pain—often described as burning or electric shock-like—represents one of the most debilitating consequences of PTNs. Its management remains complex, and for this reason, several therapeutic strategies have been proposed, ranging from pharmacological modulation of neuropathic pathways to topical treatments, with treatment selection guided by pain characteristics, severity, and individual patient response [47,48]. These alterations have a substantial impact on quality of life. Pogrel et al. report that 43% of patients experience severe difficulties with eating, 38% with speaking, and 37% develop reactive depressive symptoms [5].

The Seddon and Sunderland classifications remain clinically useful because they provide a morphologically grounded framework for estimating prognosis.

In this context, clinicians must overcome their “anchoring bias,” namely the cognitive tendency to remain attached to the expectation of spontaneous recovery, while overlooking warning signs of severe axonal injury [4]. Failure to recognize clinical progression or reliance on passive monitoring (“wait and see”) deprives the patient of the optimal therapeutic window for reparative microsurgery. Timely identification of high-grade injuries is therefore essential for early appropriate intervention, which may increase the likelihood of functional sensory recovery. Therefore, the importance of accurate diagnostic framing lies in the ability to promptly objectify nerve integrity, preventing an operative complication from consolidating into irreversible permanent injury [43].

The integration of advanced instrumental protocols represents a decisive step beyond the intrinsic limitations of subjective clinical assessment, allowing more objective characterization of neuropathy from the baseline of the injurious event through its functional stabilization. A recurrent limitation in routine clinical practice highlighted in the literature concerns the absence of an individualized pre-operative neurosensory baseline, which makes it challenging to determine the precise grading of the resulting deficit. Systematic mapping of the affected sensory territory, complemented by standardized clinical photographs documenting the distribution and extent of the sensory deficit, should be recorded in the patient’s medical baseline records for subsequent longitudinal assessment. Nevertheless, the introduction of high-field MRN may provide immediate prognostic evaluation and objective structural information during a phase in which conventional clinical assessment remains uncertain [2,49].

In parallel, monitoring the evolution of the lesion through QST may provide a longitudinal assessment of sensory recovery across different classes of nerve fibers. The accuracy of such instrumental monitoring provides greater diagnostic sensitivity than traditional chairside semiology. The complementary use of thermal QST and neurophysiological investigations such as the mental nerve blink reflex may reduce false-negative rates, confirming sensory impairment in almost all symptomatic patients [50].

To further enhance objectivity—especially in medico-legal contexts—CPT measurement using the Neurometer provides neuroselective, quantitative data independent of active patient cooperation, particularly useful when objective documentation is required [36].

The medico-legal assessment of PTNs cannot be separated from the temporal dimension, which delineates the transition from the acute injury phase to the consolidation of permanent damage. From a medico-legal perspective, this temporal gap is particularly relevant because failure or delay in diagnostic management in the postoperative period constitutes a loss of chance, depriving the patient of access to microsurgical intervention within the optimal therapeutic window that offers the highest likelihood of functional recovery.

From a forensic standpoint, the analysis does not merely capture the final clinical picture but must reconstruct the evolution of the anatomo-functional injury, including the initial deficit, subsequent clinical findings, diagnostic investigations and timing of management, to determine potential profiles of omission or delayed intervention [51]. In this context, clinicians must overcome their anchoring bias of remaining attached to the traditional “wait and see” approach, while overlooking warning signs. Although spontaneous recovery has been reported in approximately 85–96% of cases, the absence of progressive sensory improvement may indicate a less favorable prognosis. Failure to recognize this scenario or prolonged diagnostic inertia may have medico-legal implications when delayed recognition or referral results in a clinically relevant loss of chance. The concept of loss of chance, recognized in different forms across medico-legal systems, refers to the potential harm resulting from the deprivation of a patient’s therapeutic opportunity to achieve a more favorable outcome because of delayed diagnosis or treatment. For example, in Italy this principle is upheld by the Italian Supreme Court (Cass. 4400/2004 and 21619/2007), although its legal interpretation and application are jurisdiction-specific.

In this context, serial clinical and instrumental assessments (via NST, QST, and 3.0-Tesla MRN) may provide objective documentation of the evolution of the deficit and support clinical decisions regarding the transition from conservative management to surgical intervention [13,52].

Finally, medico-legal assessment should consider the functional and psychosocial impact of persistent sensory disturbances on quality of life (QoL).

Severity assessment of iatrogenic trigeminal neuropathy requires integration of patient-reported symptoms with objective clinical and instrumental findings. NST remains essential as the first-line assessment, but not sufficient for definitive severity grading.

QST and neurophysiological investigations may complement routine NST by providing a more standardized assessment of sensory function.

Objective quantitative measures, such as CPT, may be particularly valuable due to its double-blind nature, especially in medico-legal assessments when clinical findings and patient-reported symptoms are discordant.

Furthermore, MRN may provide an important structural complement to functional testing by allowing for direct assessment of the injured nerve and potentially improving differentiation between lesions with different prognostic implications. This structural information is particularly relevant in the early phase of nerve injury and when clinical findings are insufficient, potentially improving prognostic stratification.

For the lingual nerve, dystrophic signs, such as loss of fungiform papillae, may further support the clinical diagnosis of a high-grade lesion.

Overall, current evidence supports an integrated multimodal approach, in which the combination of complementary diagnostic strategies may improve the clinical framework, supporting timely clinical decision-making.

## 5. Conclusions

The evidence discussed in the present narrative review supports the view that iatrogenic PTNs should no longer be regarded as unavoidable complications to be monitored over time according to the traditional observational clinical approach, but rather as anatomo-functional entities requiring objective assessment from the earliest postoperative stages within a proactive, multimodal strategy.

Conventional NST remains an important component of the initial clinical assessment, but its limitations in objectively characterizing the severity and extent of nerve injury should be recognized. Complementary quantitative and neurophysiological approaches may provide additional functional information, while MRN offers direct structural assessment of the injured nerve. In particular, MRN, through RSI, may have prognostic value, although further studies are required to strengthen the currently available evidence.

The timing of diagnosis and management remains a clinically relevant aspect of PTNs. Available evidence suggests that earlier identification of severe nerve injuries may facilitate appropriate referral and consideration of microsurgical treatment within the period during which functional recovery is more likely. However, the available evidence regarding the relationship between treatment delay and functional sensory recovery should be interpreted with appropriate caution.

From a medico-legal perspective, objective documentation of the initial neurological status, subsequent clinical evolution, diagnostic investigations, and timing of management may provide an important record of the patient’s clinical course. Concepts such as loss of chance and professional liability are jurisdiction-dependent and should therefore be interpreted according to the applicable legal framework.

Overall, modern management of iatrogenic trigeminal nerve injuries should rely on an integrated approach, combining clinical assessment, quantitative and neurophysiological testing, and advanced imaging when appropriate. Such a multimodal strategy may improve early characterization of nerve injury, support prognostic assessment, and facilitate timely and evidence-informed clinical decision-making.

## Figures and Tables

**Table 1:** Anatomical, biological and clinical features of the inferior alveolar nerve and lingual nerve.

	Inferior Alveolar Nerve	Lingual Nerve
Anatomical Course	Within mandibular bony canal	Submucosal course, lacking bony protection
Main Mechanism of Injury	Compression or traction (dental roots, implants); chemical injury (through extrusion of endodontic materials)	Local anesthesia injection and direct needle trauma; stretch during flap retraction
Primary clinical deficits	Sensory loss affecting lower lip, chin, and ipsilateral buccal gingiva	Impairment of speech, mastication, gustatory function
Spontaneous recovery tendency	High in neuropraxia and mild axonotmesis	Less favorable in Sunderland IV–V
Functional Prognosis	Favorable in low-grade injuries; reduced in severe axonal damage	Less favorable, particularly in high-grade injuries

**Table 2:** Diagnostic workup for trigeminal nerve injury assessment.

Diagnostic Method	Purpose	Function Assessed
Clinical Neurosensory Testing (NST)	Clinical grading of sensory deficits	Somatosensory function Mechanosensory and nociceptive responses
Quantitative Sensory Testing (QST)	Quantitative psychophysical method of sensory function	A-δ and C fibers function Thermal and mechanical sensory thresholds
Current Perception Threshold (CPT)	Quantitative neuroselective assessment of sensory nerve function	Electrical sensory thresholds across different nerve-fiber populations
Magnetic Resonance (3T)	Direct visualization of peripheral nerve integrity	Structural nerve damage Signal abnormalities
Somatosensory evoked potentials (SSEP)	Neurophysiological assessment of sensory pathway conduction	Somatosensory signal transmissions

## Data Availability

No new data were created or analyzed in this study. Data sharing is not applicable to this article.
